# The effects of 12 weeks of static stretch training on the functional, mechanical, and architectural characteristics of the triceps surae muscle–tendon complex

**DOI:** 10.1007/s00421-021-04654-z

**Published:** 2021-03-09

**Authors:** Stefano Longo, Emiliano Cè, Angela Valentina Bisconti, Susanna Rampichini, Christian Doria, Marta Borrelli, Eloisa Limonta, Giuseppe Coratella, Fabio Esposito

**Affiliations:** 1 Department of Biomedical Sciences for Health, Università Degli Studi di Milano, Via G. Colombo 71, 20133 Milan, Italy; 2 IRCCS Istituto Ortopedico Galeazzi, Via R. Galeazzi 4, 20161 Milan, Italy; 3 Department of Internal Medicine, The University of Utah, Salt Lake City, UT USA; 4 Geriatric Research, Education, and Clinical Centre, Veterans Affairs Medical Centre, Salt Lake City, UT USA

**Keywords:** Stretching, Flexibility, Muscle architecture, Stiffness, Electromechanical delay, Pennation angle

## Abstract

**Purpose:**

We investigated the effects of 12 weeks of passive static stretching training (PST) on force-generating capacity, passive stiffness, muscle architecture of plantarflexor muscles.

**Methods:**

Thirty healthy adults participated in the study. Fifteen participants (STR, 6 women, 9 men) underwent 12-week plantarflexor muscles PST [(5 × 45 s-on/15 s-off) × 2exercises] × 5times/week (duration: 2250 s/week), while 15 participants (CTRL, 6 women, 9 men) served as control (no PST). Range of motion (ROM), maximum passive resistive torque (PRT_max_), *triceps surae* architecture [fascicle length, fascicle angle, and thickness], passive stiffness [muscle–tendon complex (MTC) and muscle stiffness], and plantarflexors maximun force-generating capacity variables (maximum voluntary contraction, maximum muscle activation, rate of torque development, electromechanical delay) were calculated Pre, at the 6th (Wk6), and the 12th week (Wk12) of the protocol in both groups.

**Results:**

Compared to Pre, STR ROM increased (*P* < 0.05) at Wk6 (8%) and Wk12 (23%). PRT_max_ increased at Wk12 (30%, *P* < 0.05), while MTC stiffness decreased (16%, *P* < 0.05). Muscle stiffness decreased (*P* < 0.05) at Wk6 (11%) and Wk12 (16%). No changes in *triceps surae* architecture and plantarflexors maximum force-generating capacity variables were found in STR (*P* > 0.05). Percentage changes in ROM correlated with percentage changes in PRT_max_ (*ρ* = 0.62, *P* = 0.01) and MTC stiffness (*ρ* = − 0.78, *P* = 0.001). In CTRL, no changes (*P* > 0.05) occurred in any variables at any time point.

**Conclusion:**

The expected long-term PST-induced changes in ROM were associated with modifications in the whole passive mechanical properties of the ankle joint, while maximum force-generating capacity characteristics were preserved. 12 weeks of PST do not seem a sufficient stimulus to induce *triceps surae* architectural changes.

## Introduction

Passive stretching is widely performed in sport and rehabilitation mainly to improve joint range of motion (ROM) and muscle performance. However, evidence exists that passive stretching, when performed acutely, may induce negative changes in muscle function, such as a reduced force-generating capacity (Power et al. 2004; Kay and Blazevich 2012; Longo et al. 2014; Behm et al. 2016; Trajano et al. 2017; Cè et al. 2020) and a depressed rate of torque development (RTD) (Simic et al. 2013; Trajano et al. 2019). These alterations are often accompanied by a reduction in the amplitude of the surface electromyographic (sEMG) signal from the contracting muscle after stretching (Behm et al. 2001, 2016; Cramer et al. 2005). These impairments may be ascribed to both neuromuscular, such as a reduced activation (Behm et al. 2016; Trajano et al. 2017), and mechanical mechanisms, such as alteration in the viscoelastic properties of the muscle–tendon complex (MTC) (Magnusson et al. 1996; Morse et al. 2008; Longo et al. 2014).

Whether or not a long-term (chronic) passive stretching training (PST) could affect muscle function still remains controversial. On one side, indeed, some studies reported an increase in maximum voluntary dynamic (Kokkonen et al. 2007; Nelson et al. 2012) and isometric (LaRoche et al. 2008) contraction, as well as a reduction in whole-joint (Kubo et al. 2002; Guissard and Duchateau 2004; Nakamura et al. 2012) and muscle stiffness (Blazevich et al. 2014; Nakamura et al. 2020) after chronic stretching. On the other side, other works reported no effects of PST on maximum muscle strength (Akagi and Takahashi 2014; Konrad and Tilp 2014; Blazevich et al. 2014; Sato et al. 2020), amplitude of sEMG detected during maximum voluntary force production (Blazevich et al. 2014) and whole-joint (Konrad and Tilp 2014; Blazevich et al. 2014) and muscle stiffness (Konrad and Tilp 2014). This apparent discrepancy could be justified by differences in methodological approach, PST duration, number and duration of weekly stretching sessions, and stretch intensity (Freitas et al. 2018).

After PST, architectural adaptations (fascicle length, fascicle angle, and muscle thickness) in the stretched muscle are still a matter of debate (Medeiros and Lima 2017; Nunes et al. 2020). Since changes in these variables would influence muscle contraction characteristics (Narici et al. 2016), it may be of great interest to investigate the potential PST-induced architectural adaptations. Animal studies showed that chronically stretched muscles undergo an increase in fibre size/muscle mass and fibre length (Sola et al. 1973; Holly et al. 1980; Barnett et al. 1980), possibly due to mechanical signalling-induced increase in protein synthesis and addition of series sarcomeres (Goldspink et al. 1995, 2002). Moreover, regional differences seem to occur in the chronically stretched muscle, since a different remodelling was observed between its middle and distal portions (Dix and Eisenberg 1990). However, the architectural responses to PST in humans are not clear (Nakamura et al. 2012; Akagi and Takahashi 2014; Konrad and Tilp 2014; Blazevich et al. 2014; Freitas and Mil-Homens 2015; Simpson et al. 2017; Sato et al. 2020; Beltrão et al. 2020) and more studies may be required to evaluate the impact of a long-term PST program on muscle architecture (Medeiros and Lima 2017).

Therefore, the aims of this study was to assess whether or not changes in ROM induced by long-term PST are accompanied by changes in muscle force-generating capacity, passive whole-joint and muscle stiffness, and architectural MTC characteristics. The possible correlations between changes in ROM and changes in force-generating capacity, passive stiffness, and architectural variables were also examined. Hypothesis was made that, with sufficient training volume, PST could reduce passive stiffness while increasing fascicle length and muscle thickness. Therefore, the reduction in passive stiffness would impair force transmission to the tendon insertion point, thus reducing the muscle force-generating capacity and RTD. Nevertheless, the hypothesised modifications in architectural characteristics would lead to an increase in force production. The net effect of these two balancing mechanisms would result into unchanged maximum force expression.

## Methods

### Participants

Based on pilot testing, the sample size was computed using statistical software (G-Power 3.1, Dusseldorf, Germany) expecting at least a *moderate* Cohen’s *d* effect size (0.60) in ROM changes. Considering α = 0.05 and a required power (1 − β) = 0.80, the desired sample size resulted in 24 participants. To ensure sufficient statistical power, 30 healthy volunteers (12 women) were enrolled in the study (mean ± standard deviation: age 22.7 ± 1.8 years, body mass 68.4 ± 9.4 kg, stature 1.74 ± 0.1 m), and were randomly assigned to a stretching training group (STR; *N* = 15, 6 women, 9 men; age 22.3 ± 0.8 years, body mass 68.5 ± 9.4 kg, stature 1.74 ± 0.08 m) and a control group (CTRL: *N* = 15, 6 women, 9 men; age 23.4 ± 0.8 years, body mass 67.4 ± 9.5 kg, stature 1.73 ± 0.08 m) with the same number of women in each group. Each participant received a full explanation of the aim of the study, the experimental procedures, and signed a written informed consent. The volunteers were recreationally active university students engaged in regular sports activities (2.0 ± 1.0 h/week). Exclusion criteria were: (i) the presence of musculoskeletal injury within the past 6 months; (ii) neurological deficit affecting their ability to stretch; (iii) being regularly involved in stretching training; and (iv) the presence of all those circumstances in which stretching training is contraindicated (e.g., joint and tissue laxity). Participants were asked to abstain from ergogenic beverages or similar in the 24 h preceding the test sessions and to report to the laboratory without any form of heavy intensity physical exercise in the previous 48 h. The study was approved by the local University Ethical Committee (CE 27/17) and had been performed in accordance with the latest principles of the Declaration of Helsinki.

### Experimental design

A randomised pre–post parallel group design was adopted to study the effects of a 12-week PST programme on the functional, mechanical and architectural properties of the *triceps surae* MTC. The study lasted 15 weeks. In the first two weeks, participants underwent two familiarisation sessions interspersed by at least 48 h to get acquainted with all testing procedures. A third session was attended to collect baseline data (Pre). Subsequently, participants enrolled in the STR group were instructed about the exercises to be performed on the dominant limb (right for all participants) included in the training programme and familiarised with the exercise intensity. Thereafter, STR started the 12 weeks of PST, whereas participants in CTRL continued their habitual activities. All individuals were tested at the 6th week (Wk6) and at the 12th week (Wk12) of the protocol. In males, the last evaluation occurred within a week from the end of the PST in STR. To minimise the effects of the menstrual cycle on the assessments, female participants recorded their menstrual cycle in a personal diary at the beginning and throughout the study. Knowing the occurrence of the early follicular phase allowed women to be tested around the same menstrual days (3 ± 3 days from the early follicular phase). However, this choice implied that tests may have occurred with a ± 5 day-dispersion from the exact testing week at Wk6 evaluation (Bisconti et al. 2020).

**Fig. 1:**
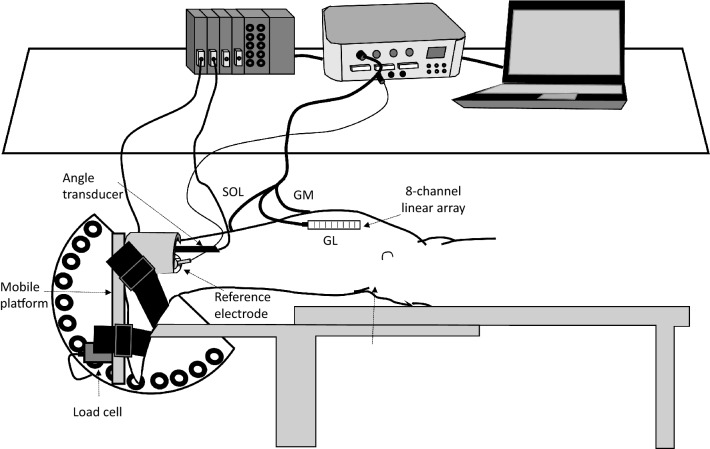
Schematic representation of the experimental set-up, showing the 8-channel linear arrays together with the reference electrode for surface electromyogram detection on the *gastrocnemius medialis* (GM), *gastrocnemius lateralis* (GL) and *soleus* (SOL) muscles, the angle transducer, and the load cell for force detection

All experiments were carried out in a room at constant temperature (22 ± 1 °C) and relative humidity (50 ± 5%). During tests, participants lay prone on a custom-made ergometer (Fig. 1) (Longo et al. 2017), with a mobile metal platfrom for consistency with Fig. 1 connected to a previously calibrated load cell (mod. SM-2000 N, Interface, UK; operating linearly between 0 and 2000 N). The ankle of the dominant limb was firmly attached to the mobile metal plate by a Velcro^®^ strap (Velcro Industries Inc., Willemstad, Netherlands Antilles) to minimize heel displacement during assessments. Hip and shoulders were also firmly secured to the ergometer. The load cell was constantly kept in line with the axis of force. Force signal was driven to an A/D converter (mod. UM 150, Biopac, Biopac System Inc., Santa Barbara, CA, USA), sampled at 10,240 Hz, directed to an auxiliary input of the electromyography amplifier (mod. EMG-USB, OtBioelettronica, Turin, Italy) and stored on a personal computer. Torque was calculated by multiplying the force output by the distance between the apical aspect of the external malleolus and the force application point. A previously calibrated bi-axial angle transducer (mod. TSD 130A, Biopac System, CA, USA) was positioned on the external face of the fibula and on the calcaneum to monitor the changes in ankle ROM.

First, ultrasound images of the *triceps surae* muscle were obtained at rest with the ankle joint fixed at 0° (perpendicularity between the tibia and the longitudinal axis of the foot). Thereafter, maximum ROM together with passive force exerted during the manoeuvre was evaluated. Passive MTC mechanical properties were subsequently assessed by combining passive force and ultrasound techniques. Lastly, the foot was re-positioned at 0° to determine the peak isometric torque. Myoelectric activity was detected during passive movement and muscle contraction.

### Measurements and data analysis

#### Ultrasound imaging

Since previous studies showed that the different *triceps surae* muscles can undergo different strain during stretch exercise (Hirata et al. 2016) and within the same muscle different portions can be unequally affected by the stretch manoeuvre (Andrade et al. 2020), we examined the possible PST-induced architectural adaptations in all *triceps surae* heads at different regions.

Muscle architecture was assessed in vivo at rest for *gastrocnemius medialis* (GM), *gastrocnemius lateralis* (GL) and *soleus* (SOL) muscles by B-mode ultrasound (LOGIQS7, GE©, Fairfield, Connecticut, USA) with a 5-cm linear-array probe (mod. 9L, 3.1–10.0 MHz) in extended-field-of-view mode (LOGIQ_view_). The participants lay prone on the examination bed with hip and knee joint extended and the ankle fixed at 0°. Transmission gel was applied to improve acoustic coupling. All muscles were inspected before extended-field-of-view acquisition. The proximal and distal ends (i.e., myotendinous junction – MTJ) of each head of the *triceps surae* were identified by moving the ultrasound probe along the longitudinal axis of each muscle belly. Thereafter, each muscle was marked on the skin at 50% length. At this location, medial and lateral muscle boundaries were also identified for each muscle, and the 50% width was drawn on the skin. A single snapshot was collected at this site for each muscle to obtain muscle thickness (MT). Subsequently, after ultrasound inspection, the medial and lateral GM and GL muscle boundaries were drawn from their respective MTJ along muscle length. Then, the probe was moved from each MTJ towards the proximal end of the respective muscle belly. When necessary, transducer manipulation occurred so that the fascicles and both aponeuroses remained continuous and visible. This path was assumed to be the best fascicle plane that could be followed, and it was marked on the skin (Franchi et al. 2020). After the inspection, a continuous single view was taken for GM and GL heads starting from the MTJ and by moving slowly the probe along the drawn line ensuring that the extended-field-of-view image exceeded the 50% muscle length. Due to its short fascicles, SOL images were captured as single snapshots at the MTJ and mid-belly sites. The expert operator performing ultrasound scanning ensured minimal pressure was applied.

**Fig. 2:**
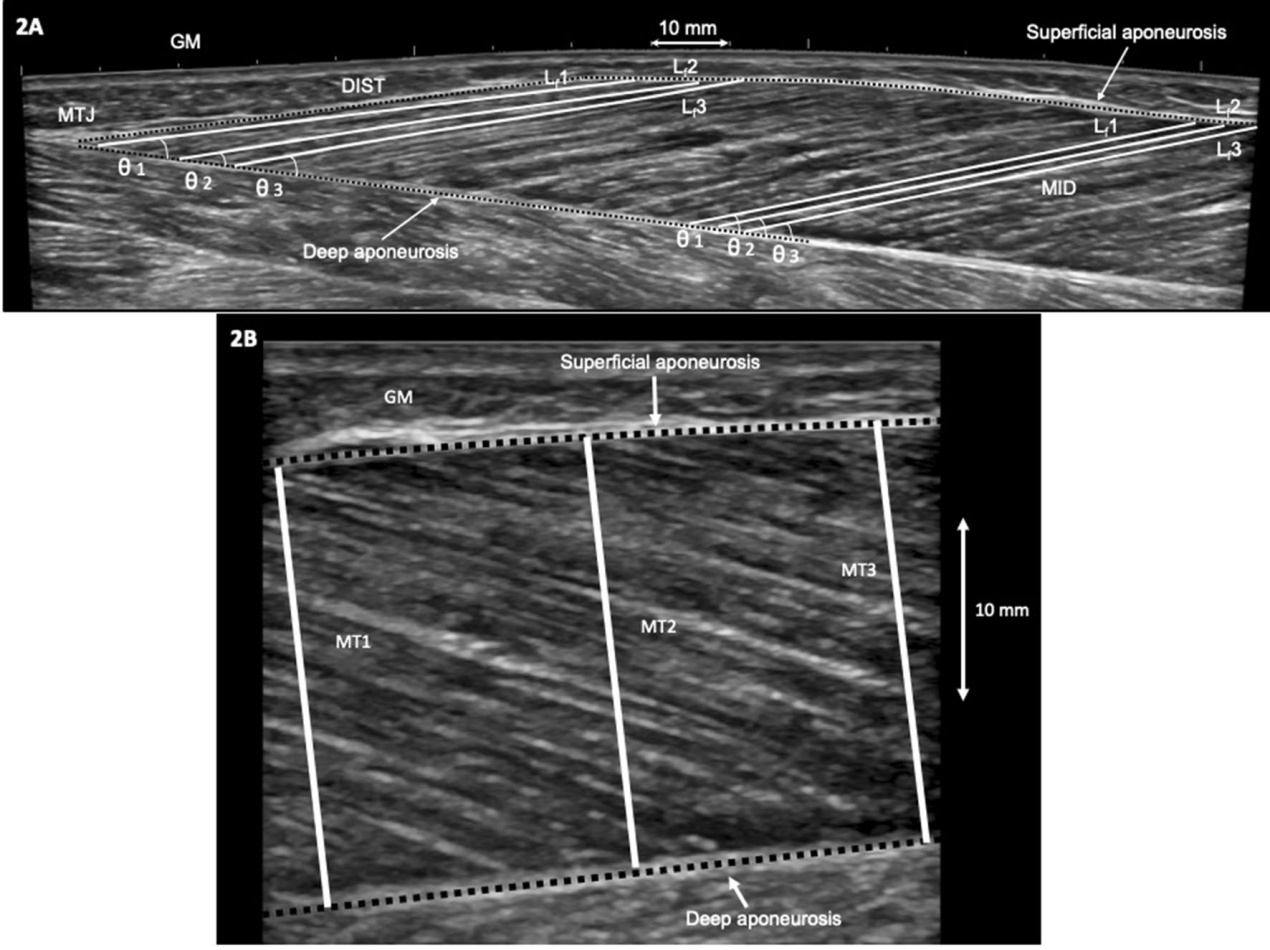
Sagittal plane ultrasound images showing examples of muscle architecture measurements of the *gastrocnemius medialis* (GM) muscle. **a** GM extended field-of-view image with fascicle length (*L*_f_) and fascicle angle (*θ*) determination at middle (MID) and distal (DIST) portions of the muscle. For each site, three fascicles (*L*_f_1, *L*_f_2, and *L*_f_3) and their respective angles (*θ*1, *θ*2, and *θ*3) were identified and measured. *MTJ* muscle–tendon junction. **b** GM single snapshot in MID showing the three sites at which muscle thickness (MT) was measured (MT1, MT2, and MT3) as the perpendicular distance between the superficial and deep aponeuroses

The images were analysed offline using an open-source computer program (ImageJ 1.44b, National Institutes of Health, USA). For each muscle, three clearly visible muscle fascicles were identified at 50% muscle length (MID) and close to the MTJ (DIST—i.e., about 2 cm along the deep aponeurosis from the MTJ) (Fig. 2a). Muscle fascicle length (*L*_f_) was measured by drawing a line along the three fascicles between the deep and superficial aponeurosis. Any fascicle curvature was taken into account. On the same highlighted fascicles, their insertion angle into the deep aponeurosis was measured as fascicle angle (*θ*). The three measured fascicles and angles were averaged and used for the analysis. MT of each *triceps surae* head was measured as the distance between the superficial and deep aponeurosis at three points where the muscle belly was the widest and the two aponeuroses parallel (Fig. 2b) from the single snapshot taken at MID. These three measures were then averaged.

#### ROM and maximum passive resisting torque

As described in the “Experimental design” section, the ankle was securely fixed to the custom-made ergometer for ROM and maximum passive resisting torque (PRT_max_) detection. After 10 conditioning passive ankle movements, the joint ROM was determined starting with the ankle at its resting position (~ − 20° of dorsiflexion) and manually dorsiflexed at slow speed to avoid reflex activations monitored by surface electromyography (sEMG) until the participant’s point of discomfort was reached. At maximum ROM, the ankle joint was mechanically fixed for the recording of PRT_max_, which was considered as index of stretch tolerance (Kay et al. 2016).

The difference between the ankle at 0° and the end ROM was considered as joint ROM. Reliability was previously reported (intraclass correlation coefficient—ICC = 0.94; standard error of measurement as percentage—SEM% = 1.1) (Longo et al. 2014). PRT_max_ was measured within a 250-ms epoch (Kay et al. 2016).

### Passive MTC and GM muscle stiffness

To allow measurements of passive force and GM MTJ position at different joint angles, the metal plate was manually fixed at 0°, 10°, 20° of ankle dorsiflexion, and at end ROM (Longo et al. 2014). Joint positioning was executed at slow speed to avoid reflex muscle activation (monitored by sEMG). PRT exerted by plantarflexor muscles was recorded at each angle as the average of force values during the first 5 s after ankle positioning. Such a short time allowed the operator to minimize the influence of the static position on the MTC viscoelastic properties. The passive torque–angle curve between 0° and 20° of dorsiflexion was fitted with the best polynomial regression model (Mizuno et al. 2013; Longo et al. 2014), and the slope of this curve at 20° of dorsiflexion (maximum common angle for all participants) represented passive MTC stiffness.

While passive MTC stiffness was assessed, GM MTJ displacement was also captured by means of B-mode ultrasound imaging using a 5-cm linear-array probe. A hypoechoic tape was applied on the skin close to the MTJ for monitoring possible shifting during measurement. GM MTJ was visualised in a continuous sagittal plane at 0°, 10°, 20° of dorsiflexion, at end ROM, and digitised offline relative to the hypoechoic marker. As previously described (Morse et al. 2008; Cè et al. 2015), possible artefacts due to skin shift during MTJ displacement assessment were corrected accordingly.

GM muscle stiffness was calculated by dividing the changes in PRT between 0° and 20° by the corresponding MTJ displacement. MTJ displacement between 0° and end ROM (ΔMTJ_max_) represented maximum GM elongation.

Reliability analysis for passive MTC (ICC = 0.91, SEM% = 2.4), and GM muscle stiffness (ICC = 0.93, SEM% = 4.1) was reported previously (Longo et al. 2014).

#### Maximum voluntary contraction

After a standardized warm-up (10 × 2-s contractions of increasing intensity from 50% maximum voluntary contraction determined during familiarization up to maximum), plantarflexors maximum voluntary isometric contraction (MVC) was assessed at 0° (anatomical neutral position). Participants were instructed to contract “as fast and hard as possible” (Maffiuletti et al. 2016), to maintain the contraction for 3 s and then to relax. Two trials were performed separated by 3 min of rest. In case of a between-trial difference > 5%, a third trial was executed. The best trial was used as MVC.

#### Electromyographic assessment

sEMG signal was collected during MVC measurements in GM, GL and SOL. The myoelectric activity was detected by a linear array of eight electrodes (mod. KITAD008, OtBioelettronica, Turin, Italy; probe: 45 mm × 20 mm; electrode length: 2 mm; inter-electrode distance: 5 mm) fixed to the skin by dual-adhesive foams (mod. AD004, OtBioelettronica, Turin, Italy) and filled with conductive gel (Cogel, Comedical, Trento, Italy). The skin area under the sEMG electrodes was cleaned with ethyl alcohol, abraded gently with fine sandpaper and prepared with a conductive cream (Nuprep, Weaver and Co., Aurora, USA) to achieve an inter-electrode impedance below 2000 Ω. For each muscle, the sEMG array was placed over the muscle belly along the direction of the muscle fibres, in accordance with the European recommendations for surface EMG (Hermens et al. 1999). sEMG was acquired by a multichannel amplifier with a sampling rate of 10,240 Hz (mod. EMG-USB, OtBioelettronica, Turin, Italy; input impedance: > 90 MΩ; CMRR: > 96 dB), amplified (gain × 1000) and filtered (filter type: 4th order Butterworth filter; bandwidth: 10–500 Hz).

The analysis was performed by OtBiolab+ software (OtBioelettronica, Turin, Italy). The sEMG signal epochs were aligned with the same force signal epochs. The sEMG signal of each muscle was analysed in time domain within the same 1-s period detected in the middle of the MVC plateau. The sEMG root mean square (RMS) was calculated in consecutive 250-ms time windows and then averaged.

#### RTD

Rate of torque development was measured using the raw force trace while assessing MVC. A threshold of three standard deviations above the baseline signal for three consecutive points within a 100-ms interval of the resting condition immediately preceding the contraction was used as the onset of force signal. Due to large variability in the early time window, rate of torque development was calculated as the average change in torque per time interval from the torque onset to 200 ms (RTD_200_) (Maffiuletti et al. 2016; Trajano et al. 2019). Reliability for RTD has been previously reported (ICC = 0.91, SEM% = 6.5) (Longo et al. 2014).

#### Electromechanical delay

The electromechanical delay (EMD) was calculated as the time between the onset of the band-passed, rectified GM sEMG signal and torque onset during MVC (Longo et al. 2017). The same criteria for torque onset identification (three standard deviations above the baseline signal preceding the contraction) were used for detecting sEMG onset. Reliability analysis for EMD was reported previously (ICC = 0.97, SEM% = 1.1) (Longo et al. 2017).

#### PST programme

**Fig. 3:**
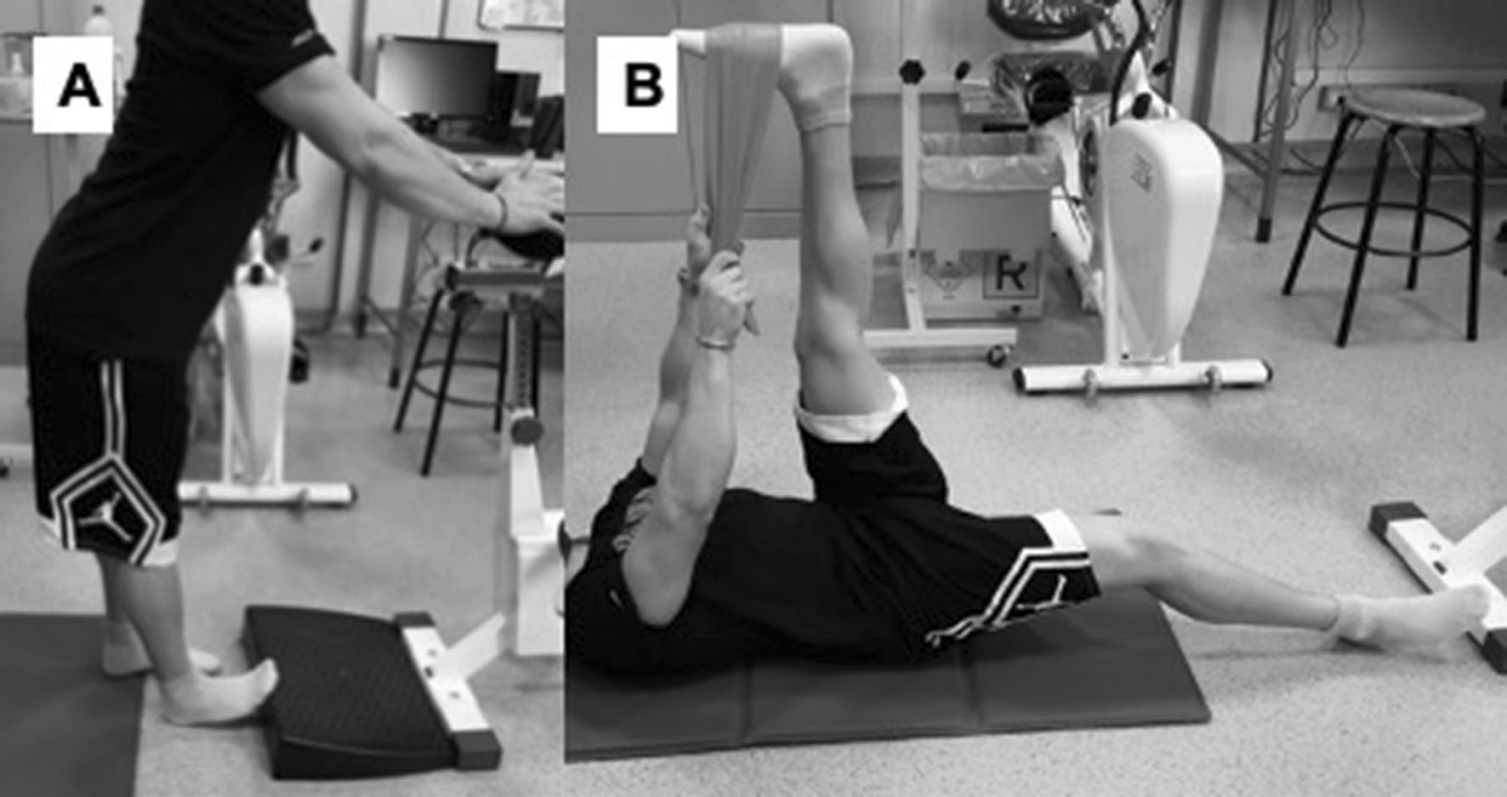
Each exercise comprises a set of five stretches of 45 s with 15 s of rest in between. The exercises were performed by the stretching training group. **a** Ankle dorsiflexion in orthostatic position; **b** hip flexion + ankle dorsiflexion with straight leg in supine position

The PST programme consisted of 12 weeks of training, 5 sessions *per* week (60 sessions in total). This choice was based on recent studies pointing out that investigations providing the participants with an adequate training volume (i.e., weekly frequency > 3 times/week, total duration > 8 weeks) were needed to clarify the effects of PST on the functional, mechanical and architectural characteristics of the MTC (Freitas et al. 2018; Nakamura et al. 2020; Andrade et al. 2020). Each session included two manoeuvres for plantarflexor muscles (Fig. 3), 45 s elongation and 15 s recovery in the starting position repeated for five times (Longo et al. 2014). The exercises were performed on the right limb and stretching duration was 450 s each session, giving a total stretching time of 2250 s *per* week. For the first exercise, the participants stood erect with arms supporting the body and the right foot in dorsiflexion against a board (Fig. 3a). For the second exercise, the participants lay supine starting with the right knee straightened and an elastic band around the right foot positioned at maximum dorsiflexion. Using the arms while holding the elastic band, the right hip was flexed until maximum point of discomfort (Fig. 3b). The exercises were chosen to represent a stretch modality commonly performed in recreational (e.g., gyms) and sports activities. As stated above, the CTRL group received no PST. To promote participants’ compliance, daily classes were held at different day time (morning and afternoon) at the University Sports Centre gym. Each class was supervised by an expert operator, which monitored the attendance, the correct exercise execution, and the intensity exerted during the exercise. Participants were instructed to stretch around the maximally tolerable stretch within the pain limit (Blazevich et al. 2014; Cè et al. 2020). Individuals not attending at least the 80% of classes were excluded from the study, and new participants were recruited to substitute the drop out.

### Statistical analysis

Statistical analysis was performed using a statistical software package (IBM SPSS Statistics v. 26, Armonk, NY, USA). The Shapiro–Wilk test was used to check the normal distribution of the sampling. To determine possible baseline differences between STR and CTRL before intervention, the unpaired Student’s *t* test was applied for each variable of interest. A two-way mixed-model analysis of variance (ANOVA) [within-group factor: time, 3 levels (Pre, Wk6, Wk12); between-groups factor: group, 2 levels (STR, CTRL)] was used to check for differences between groups over time. To assess between-groups differences in the changes over time, an analysis of co-variance (ANCOVA) was applied, using values at Pre as covariate. Multiple comparisons were perfomed applying the Bonferroni’s correction. For every variable of interest, the percentage variation was calculated for each participant at each time point. Thereafter, the mean percentage difference of the sample with 95% confidence interval (95% CI) was calculated. Since the Pre-Wk12 percentage changes in PRT_max_ and GM muscle stiffness of the STR group were not normally distributed, the possible correlations between percentage changes in ROM and PRT_max_, MTC stiffness, and GM muscle stiffness were assessed by Spearman’s Rho coefficient.

The ANOVA effect size was evaluated with partial eta squared (*η*_p_^2^) and classified as follows: < 0.06: *small*; if, 0.06–0.14: *medium*; and > 0.14: *large* (Cohen 1988).The Hedge’s *g* effect size with 95% CI was also calculated and interpreted as follows: 0.00–0.19: *trivial*; 0.20–0.59: *small*; 0.60–1.19: *moderate*; 1.20–1.99: *large*; ≥ 2.00: *very large* (Hopkins et al. 2009). Data are presented in mean ± SD. Statistical significance was set with *P* < 0.05.

## Results

### Participants’ compliance

Attendance was about 90% (54/60 training sessions). Four participants dropped out because of personal reasons not linked to the study. They were immediately replaced to maintain the sample size.

### ROM and PRT_max_

ROM values are presented in Table 1. There was no significant difference between groups at Pre (*t* = − 0.10, *P* = 0.92; *g* = 0.04, *trivial*). As expected, the ANOVA revealed a main effect for time (*P* < 0.01, *large*) and interaction (*P* < 0.01, *large*). In STR, ankle dorsiflexion ROM increased by 8.8% at Wk6 compared to Pre (95% CI = 4.4–13.2%, *P* < 0.01; *g* = 0.59, *small*, 95% CI = − 0.14–1.32), and subsequently increased by 13.2% at Wk12 (95% CI = 7.1–19.3%, *P* < 0.01; *g* = 1.12, *moderate*, 95% CI = 0.35–1.89). The Pre-Wk12 change was 23.4% (95% CI = 14.0–32.7%, *P* < 0.01; *g* = 1.63, *large*, 95% CI = 0.81–2.46). ROM did not change significantly in CTRL at any time point (Pre-Wk12: 7.7%, 95% CI = − 0.07–14.2%, *P* = 0.46; *g* = 0.26, *small*, 95% CI = − 0.46–0.98, Table 1). The ANCOVA revealed a significant difference between STR and CTRL (*P* < 0.01) at Wk12.

**Table 1:** Range of motion (ROM), maximum passive resistive torque (PRT_max_), and plantarflexor muscles force-generating capacity variables before (Pre), at the 6th week (Wk6), and at the 12th week (Wk12) of study protocol in both passive stretching training (STR) and control (CTRL) groups

Parameter	STR (*N* = 15)			CTRL (*N* = 15)			ANOVA	
	Pre	Wk6	Wk12	Pre	Wk6	Wk12	Time effect	Group × time interaction
ROM (°)	23.3 ± 3.3	25.5 ± 3.5^a^	28.3 ± 3.0^a,b,^**	23.5 ± 4.0	24.3 ± 3.6	24.6 ± 4.0	*F* = 26.90 *P* < 0.01 *η*_p_^2^ = 0.49	*F* = 10.75 *P* < 0.01 *η*_p_^2^ = 0.28
PRT_max_ (Nm)	39.4 ± 9.9	46.4 ± 14.8	49.3 ± 15.3^a,^*	41.9 ± 9.4	41.4 ± 9.9	41.2 ± 10.4	*F* = 3.66 *P* = 0.03 *η*_p_^2^ = 0.12	*F* = 4.85 *P* = 0.01 *η*_p_^2^ = 0.15
MVC (Nm)	147.2 ± 32.1	148.7 ± 32.4	150.4 ± 32.6	151.7 ± 33.7	152.8 ± 32.8	153.9 ± 38.1	*F* = 1.36 *P* = 0.26 *η*_p_^2^ = 0.03	*F* = 0.05 *P* = 0.95 *η*_p_^2^ = 0.001
GM sEMG (mV)	0.47 ± 0.08	0.49 ± 0.09	0.48 ± 0.09	0.48 ± 0.10	0.50 ± 0.09	0.49 ± 0.10	*F* = 1.37 *P* = 0.26 *η*_p_^2^ = 0.03	*F* = 1.52 *P* = 0.22 *η*_p_^2^ = 0.04
GL sEMG (mV)	0.39 ± 0.09	0.39 ± 0.08	0.38 ± 0.10	0.38 ± 0.10	0.37 ± 0.08	0.39 ± 0.09	*F* = 0.33 *P* = 0.72 *η*_p_^2^ = 0.01	*F* = 1.22 *P* = 0.30 *η*_p_^2^ = 0.03
SOL sEMG (mV)	0.28 ± 0.05	0.29 ± 0.05	0.27 ± 0.07	0.28 ± 0.08	0.27 ± 0.08	0.29 ± 0.07	*F* = 0.31 *P* = 0.74 *η*_p_^2^ = 0.01	*F* = 1.80 *P* = 0.17 *η*_p_^2^ = 0.05
RTD_200_ (Nm/s)	364.8 ± 119.6	367.0 ± 127.7	377.1 ± 125.6	386 ± 174.5	346.3 ± 188.9	344.8 ± 135.5	*F* = 1.72 *P* = 0.19 *η*_p_^2^ = 0.07	*F* = 3.02 *P* = 0.09 *η*_p_^2^ = 0.11
EMD (ms)	27.5 ± 5.1	27.8 ± 5.7	27.9 ± 4.8	26.9 ± 5.1	27.1 ± 6.0	27.3 ± 5.0	*F* = 0.11 *P* = 0.90 *η*_p_^2^ = 0.004	*F* = 0.01 *P* = 0.98 *η*_p_^2^ = 0.001

Values are mean ± SD

MVC, maximum voluntary contraction; sEMG, surface electromyogram root mean square; GM, *gastrocnemius medialis*; GL, *gastrocnemius lateralis*; SOL, soleus; RTD_200_, rate of torque development from 0 to 200 ms; EMD, total electromechanical delay; *η*_p_^2^, partial eta-squared

^a^Statistically different from Pre with *P* < 0.05

^b^Statistically different from Wk6 with *P* < 0.05

*Statistically different from CTRL at the same time point with *P* < 0.05

**Statistically different from CTRL at the same time point with *P* < 0.01

PRT_max_ values are presented in Table 1. PRT_max_ was not significantly different between groups at Pre (*t* = 1.46, *P* = − 0.71, *g* = 0.25, *small*). The ANOVA revealed a main effect for time (*P* = 0.03, *medium*) and interaction (*P* = 0.01, *large*). In STR, PRT_max_ significantly increased by 29.9% at Wk12 (95% CI = 4.4–55.4%, *P* = 0.02; *g* = 0.75, *moderate*, 95% CI = 0.01–1.49), but not at Wk6 (22.1%, 95% CI = − 4.0–48.1%, *P* = 0.09; *g* = 0.56, *small*, 95% CI = − 0.19–1.27) compared to Pre. PRT_max_ did not change significantly at any time point in CTRL (Pre-Wk12: 1.8%, 95% CI = − 7.5–4.0%, *P* = 0.58; *g* = 0.05, *trivial*, 95% CI = − 0.78–0.65). The ANCOVA revealed a significant difference between STR and CTRL (*P* = 0.02) at Wk12.

### Muscle architecture and thickness

Mean values of all parameters are presented in Table 2. No significant differences were found between groups in any variable at Pre (*t-*range = − 1.49–0.99, *P-*range = 0.14–0.88, *g*-range = 0.03–0.54, *trivial-small*). The ANOVA did not reveal any effect of time in any muscle or location (*P*-range = 0.12–0.98, *small-medium*) or interaction (*P* = 0.10–0.91, *small-medium*) (Table 2).

**Table 2:** Architectural parameters before (Pre), at the 6th week (Wk6), and at the 12th week (Wk12) of study protocol in both passive stretching training (STR) and control (CTRL) groups

Muscle architecture	STR (*N* = 15)			CTRL (*N* = 15)			Time effect	Group × Time Interaction
	Pre	Wk6	Wk12	Pre	Wk6	Wk12		
MID								
GM *L*_f_ (mm)	58.17 ± 8.32	59.06 ± 8.54	58.31 ± 6.57	57.66 ± 8.87	57.42 ± 8.16	56.50 ± 7.80	*F* = 1.85 *P* = 0.17 *η*_p_^2^ = 0.07	*F* = 2.08 *P* = 0.14 *η*_p_^2^ = 0.08
GM *θ* (°)	20.39 ± 2.48	19.87 ± 1.98	20.40 ± 2.12	21.07 ± 2.37	21.12 ± 2.22	21.05 ± 2.37	*F* = 0.24 *P* = 0.79 *η*_p_^2^ = 0.01	*F* = 1.31 *P* = 0.28 *η*_p_^2^ = 0.04
GM MT (mm)	20.11 ± 2.38	20.52 ± 2.55	20.08 ± 1.80	19.33 ± 2.46	19.21 ± 2.13	19.20 ± 2.24	*F* = 0.35 *P* = 0.70 *η*_p_^2^ = 0.01	*F* = 1.85 *P* = 0.17 *η*_p_^2^ = 0.06
GL *L*_f_ (mm)	72.09 ± 11.48	71.54 ± 11.98	75.19 ± 11.48	70.11 ± 11.34	71.83 ± 11.34	71.83 ± 11.49	*F* = 2.21 *P* = 0.12 *η*_p_^2^ = 0.07	*F* = 1.41 *P* = 0.25 *η*_p_^2^ = 0.04
GL *θ* (°)	12.58 ± 1.48	12.73 ± 1.36	12.47 ± 1.89	12.64 ± 2.58	12.65 ± 3.06	12.64 ± 2.71	*F* = 0.07 *P* = 0.93 *η*_p_^2^ = 0.002	*F* = 0.51 *P* = 0.59 *η*_p_^2^ = 0.02
GL MT (mm)	16.11 ± 2.65	16.20 ± 2.99	17.05 ± 2.32	15.07 ± 2.63	15.13 ± 2.69	15.06 ± 2.55	*F* = 0.79 *P* = 0.46 *η*_p_^2^ = 0.02	*F* = 0.86 *P* = 0.43 *η*_p_^2^ = 0.03
SOL *L*_f_ (mm)	41.48 ± 8.44	41.05 ± 8.24	40.86 ± 8.25	40.98 ± 10.44	41.56 ± 10.49	41.18 ± 10.42	*F* = 0.78 *P* = 0.46 *η*_p_^2^ = 0.03	*F* = 2.55 *P* = 0.10 *η*_p_^2^ = 0.08
SOL *θ* (°)	21.24 ± 3.78	21.06 ± 4.07	21.50 ± 3.89	22.29 ± 5.44	22.78 ± 6.00	22.93 ± 5.89	*F* = 1.40 *P* = 0.25 *η*_p_^2^ = 0.05	*F* = 1.24 *P* = 0.30 *η*_p_^2^ = 0.04
SOL MT (mm)	15.17 ± 2.78	15.45 ± 3.03	15.28 ± 2.93	14.77 ± 3.89	15.06 ± 4.19	14.76 ± 4.22	*F* = 1.50 *P* = 0.23 *η*_p_^2^ = 0.05	*F* = 0.12 *P* = 0.88 *η*_*p*_^2^ = 0.004
DIST								
GM *L*_f_ (mm)	55.52 ± 6.46	54.63 ± 9.24	55.87 ± 9.55	59.64 ± 8.39	59.31 ± 8.29	59.53 ± 8.28	*F* = 0.59 *P* = 0.56 *η*_*p*_^2^ = 0.02	*F* = 0.09 *P* = 0.91 *η*_*p*_^2^ = 0.04
GM *θ* (°)	16.41 ± 3.32	16.01 ± 2.74	16.40 ± 3.24	15.53 ± 3.47	15.98 ± 3.73	15.80 ± 3.67	*F* = 0.76 *P* = 0.47 *η*_*p*_^2^ = 0.03	*F* = 0.95 *P* = 0.39 *η*_p_^2^ = 0.03
GL *L*_f_ (mm)	61.77 ± 12.42	59.81 ± 10.25	62.00 ± 13.25	57.97 ± 9.33	58.33 ± 10.00	58.14 ± 9.60	*F* = 0.23 *P* = 0.79 *η*_p_^2^ = 0.01	*F* = 0.14 *P* = 0.87 *η*_p_^2^ = 0.008
GL *θ* (°)	10.65 ± 1.89	10.15 ± 1.20	10.08 ± 1.97	11.68 ± 2.74	11.40 ± 2.23	11.47 ± 2.42	*F* = 0.60 *P* = 0.55 *η*_p_^2^ = 0.02	*F* = 0.10 *P* = 0.90 *η*_p_^2^ = 0.003
SOL *L*_f_ (mm)	38.32 ± 7.41	38.13 ± 7.70	38.85 ± 7.87	38.93 ± 10.66	39.40 ± 10.40	39.05 ± 10.42	*F* = 0.96 *P* = 0.39 *η*_p_^2^ = 0.04	*F* = 1.09 *P* = 0.34 *η*_p_^2^ = 0.04
SOL *θ* (°)	12.38 ± 1.71	12.55 ± 1.72	12.42 ± 1.91	13.27 ± 1.49	13.16 ± 1.71	13.26 ± 1.54	*F* = 0.02 *P* = 0.98 *η*_p_^2^ = 0.001	*F* = 0.65 *P* = 0.53 *η*_p_^2^ = 0.02

Values are mean ± SD

MID, middle portion of muscle belly; DIST, distal portion of muscle belly; GM, *gastrocnemius medialis*; GL, *gastrocnemius lateralis*; SOL, soleus; *L*_f_, fascicle length; *θ*, fascicle angle; MT, muscle thickness; *η*_p_^2^, partial eta-squared

### Muscle elongation, passive MTC and GM muscle stiffness

**Fig. 4:**
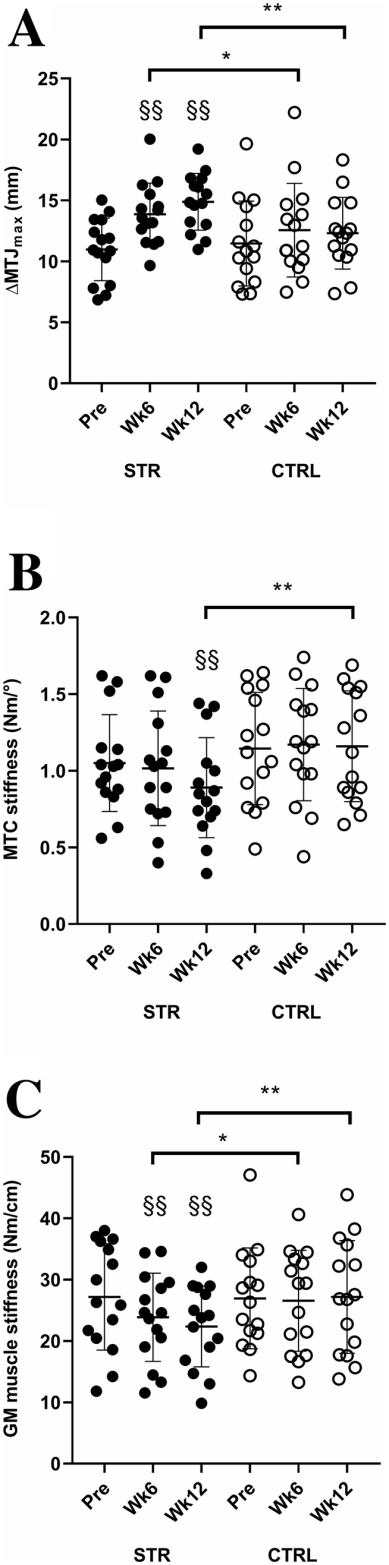
Maximum myotendinous junction elongation (ΔMTJ_max_) of *gastrocnemius medialis* (GM) muscle (**a**), passive muscle–tendon complex (MTC) stiffness (**b**), and GM muscle stiffness (**c**) in both stretching training (STR, closed circles) and control (CTRL, open circles) groups at the beginning (Pre), 6th week (Wk6), and 12th week (Wk12) of study protocol. ^§§^Significantly different from Pre with *P* < 0.01; *significantly different between groups with *P* < 0.05; **significantly different between groups with *P* < 0.01

ΔMTJ_max_ (Fig. 4a) was not significantly different between groups at Pre (*t* = − 0.43, *P* = 0.67, *g* = 0.16, *trivial*). The ANOVA revealed a main effect for time (*F* = 13.02, *P* < 0.001, *η*_p_^2^ = 0.32, *large*) and interaction (*F* = 4.72, *P* = 0.013, *η*_p_^2^ = 0.14, *medium*). In STR, muscle elongation significantly increased by 26.3% at Wk6 compared to Pre (95% CI = 17.1–42.8%, *P* = 0.01; *g* = 1.0, *moderate*, 95% CI = 0.27–1.79), followed by a further 9.8% non-significant increase at Wk12 (95% CI = − 1.9–21.5%, *P* = 0.66; *g* = 0.38, *small*, 95% CI = − 0.34–1.10). The Pre-Wk12 change was 41.9% (95% CI = 21.4–62.4%, *P* < 0.001; *g* = 1.25, *large*, 95% CI = 1.52 ± 2.37). The ANCOVA revealed a significant difference between groups at both Wk6 (*P* = 0.05) and Wk12 (*P* < 0.01). ΔMTJ_max_ did not change significantly at any time point in CTRL (Pre-Wk12: 13.7%, 95% CI = − 7.4–34.9%, *P* = 1.0; *g* = 0.24, *small*, 95% CI = − 0.48–0.96).

MTC stiffness (Fig. 4b) was not significantly different between groups at Pre (*t* = − 0.76, *P* = 0.45; *g* = 0.25 *small*, 95% CI = − 0.47–0.97). The ANOVA revealed a main effect for time (*F* = 4.94, *P* = 0.01, *η*_p_^2^ = 0.15, *large*) and interaction (*F* = 5.84, *P* = 0.005, *η*_p_^2^ = 0.17, *large*). In STR, MTC stiffness decreased by 16.5% at Wk12 (95% CI = − 9.1–− 23.8%, *P* < 0.01; *g* = 0.48, *small*, 95% CI = − 0.25–1.20), but not at Wk6 (− 4.6%, 95% CI = − 13.4–4.3%, *P* = 0.10; *g* = 0.08, *trivial*, 95% CI = − 0.63–0.80) compared to Pre. The ANCOVA revealed a significant difference between groups at Wk12 (*P* < 0.01). MTC stiffness did not change significantly at any time point in CTRL (Pre-Wk12: + 3.1%, 95% CI = − 5.4–11.7%, *P* = 1.00; *g* = 0.05, *trivial*, 95% CI = − 0.77–0.66, Fig. 2b).

GM muscle stiffness (Fig. 4c) was not significantly different between groups at Pre (*t* = 0.08, *P* = 0.94; *g* = 0.03, *trivial*, 95% CI = − 0.69–0.74). The ANOVA revealed a main effect for time (*F* = 6.25, *P* = 0.004, *η*_p_^2^ = 0.18, *large*) and interaction (*F* = 6.80, *P* = 0.002, *η*_p_^2^ = 0.19, *large*). In STR, muscle stiffness decreased by 10.8% at Wk6 compared to Pre (95% CI = − 17.5–− 4.1%, *P* = 0.01; *g* = 0.42, *small*, 95% CI = − 0.42–1.13), with a further non-significant decrease by 5.5% at Wk12 (95% CI = − 12.0–1.0%, *P* = 0.36; *g* = 0.21, *small*, 95% CI = − 0.51–0.93). The Pre-Wk12 change was − 15.9% (95% CI = − 24.5–− 7.3%, *P* = 0.004; *g* = 0.61, *moderate*, 95% CI = − 0.12–1.34). The ANCOVA revealed a significant difference between groups at both Wk6 (*P* < 0.05) and Wk12 (*P* < 0.01). In CTRL, no significant changes were found at any time point (Pre-Wk12: + 1.0%, 95% CI = − 8.7–10.1%, *P* = 0.83; *g* = 0.02, *trivial*, 95% CI = − 0.74–0.70).

### MVC and sEMG RMS

Mean values of MVC and RMS are presented in Table 1. Regarding MVC, no significant difference between groups was found at Pre (*t* = − 0.43, *P* = 0.67; *g* = 0.13, *trivial,* 95% CI = − 0.58–0.85). The ANOVA did not reveal any effect of time (*P* = 0.26, *small*) or interaction (*P* = 0.95, *small*) (Table 1).

Concerning RMS, no significant difference between groups was found at Pre (GM: *t* = − 0.32, *P* = 0.75; *g* = 0.10, *trivial*, 95% CI = − 0.62–0.81; GL: *t* = 0.40, *P* = 0.69; *g* = 0.12, *trivial*, 95% CI = − 0.59–0.84; SOL: *t* = 0.33, *P* = 0.74; *g* = 0.10, *trivial*, 95% CI = − 0.62–0.82). Similar to MVC, the ANOVA did not reveal any effect of time for GM (*P* = 0.26, *small*), GL (*P* = 0.72, *small*)**,** and SOL (*P* = 0.74, *small*) or interaction for GM (*P* = 0.22, *small*), GL (*P* = 0.30, *small*), and SOL (*P* = 0.17, *small*) (Table 1).

### RTD_200_ and EMD

Mean values of both RTD_200_ and EMD are presented in Table 1. Regarding RTD_200_, no significant difference between groups was found at Pre (*t* = − 0.37, *P* = 0.72; *g* = 0.14, *trivial,* 95% CI = − 0.58–0.85). The ANOVA did not reveal any effect of time (*P* = 0.19, *small*) or interaction (*P* = 0.09, *medium*). Concerning EMD, no significant difference between group was found at Pre (*t* = 0.32, *P* = 0.75; *g* = 0.12, *trivial*, 95% CI = − 0.60–0.83). The ANOVA did not reveal any effect of time (*P* = 0.90, *small*) or interaction (*P* = 0.98, *small*).

### Correlations

**Fig. 5:**
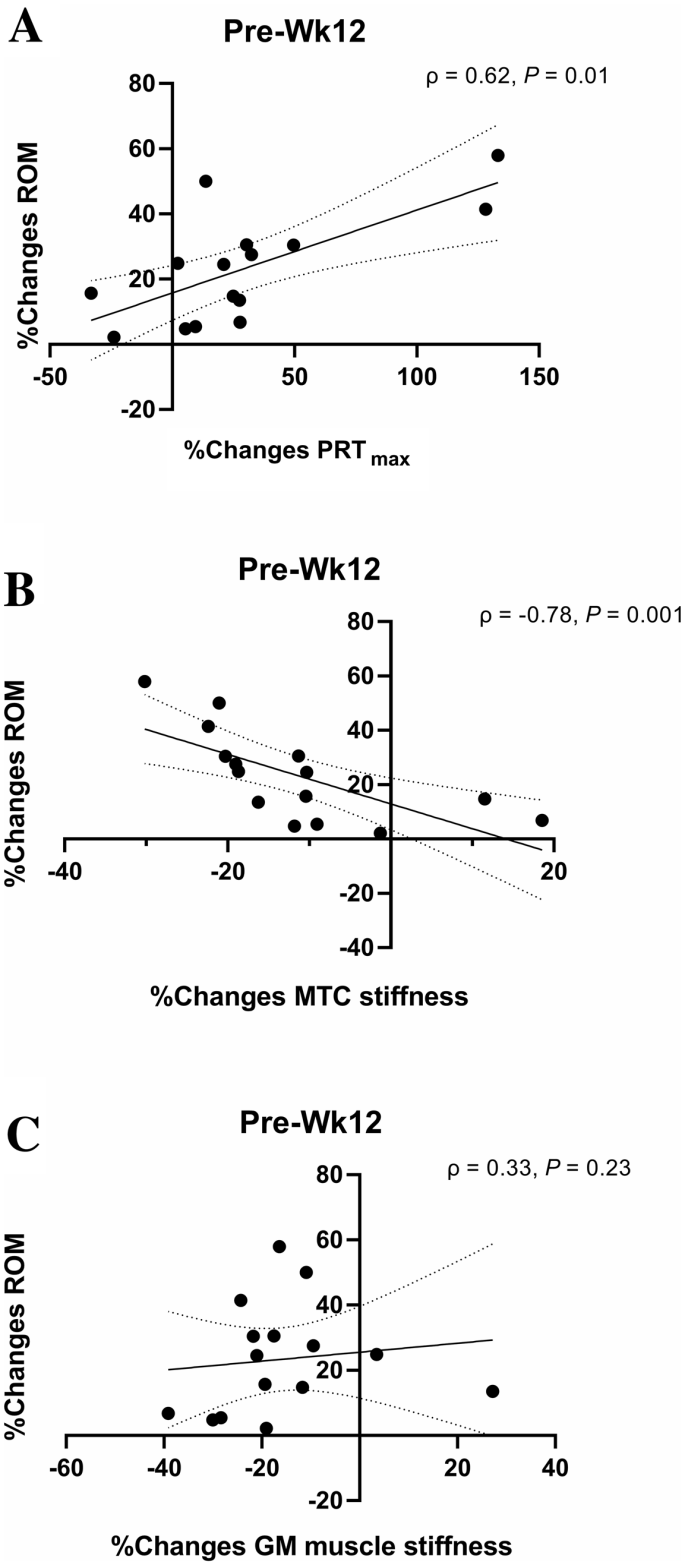
Correlations between percentage changes (%changes) in range of motion (ROM) and maximum resistive torque (PRT_max_) (**a**), passive muscle–tendon complex (MTC) stiffness (**b**), and *gastrocnemius medialis* (GM) muscle stiffness (**c**) in the stretching training group at the end of 12 weeks of training protocol (Wk12). *ρ* Spearman’s Rho coefficient

In STR, the Pre-Wk12 percentage changes in ROM significantly correlated with percentage changes in PRT_max_ (*ρ* = 0.62, *P* = 0.01, Fig. 5a), and percentage changes in MTC stiffness (*ρ* = − 0.78, *P* = 0.001, Fig. 5b), but not with percentage changes in GM muscle stiffness (*ρ* = 0.33, *P* = 0.23, Fig. 5c).

## Discussion

The main results of this investigation were that the expected PST-induced increase in ROM and PRT_max_ was accompanied by a decrease in GM muscle and whole MTC stiffness, in line with the experimental hypothesis. The percentage changes in ROM at the end of the training protocol correlated with those in PRT_max_ and MTC but not with GM muscle stiffness. Therefore, an adequate PST volume can induce whole-joint passive mechanical alterations, with GM muscle stiffness playing only a limited role in PST-induced ROM adaptations. Contrary to the experimental hypothesis, though, PST did not induce alterations in muscle architecture at any location, suggesting that this training protocol was not sufficient to induce structural adaptations detectable by ultrasound imaging technique. Interestingly, plantarflexor muscles force-generating capacity variables (MVC, sEMG RMS, RTD_200_, EMD) were not affected by PST, indicating that despite the reduction in stiffness, this training protocol did not compromise the mechanisms underpinning force transmission during MVC.

### Preliminary considerations

As expected, PST induced an increase in ankle ROM by ~ 9% at Wk6 and ~ 24% at Wk12 compared to Pre. Concomitantly, PRT_max_ (an index of stretch tolerance) increased at both time points despite being significantly higher only at Wk12 (~ 30%). A correlation was found between the percentage changes in ROM and PRT_max_ at Wk12, suggesting that one of the factors explaining the increase in ankle ROM could be attributable to the increase in stretch tolerance, in line with previous findings (Guissard and Duchateau 2004; Blazevich et al. 2014; Kay et al. 2016; Nakamura et al. 2017). Although the exact origin of this phenomenon still needs to be fully clarified, the increase in stretch tolerance could be ascribed to a change in the afferent input from nociceptive nerve endings and mechanoreceptors, as well as to the increased participants’ willingness to tolerate the greater loading at ROM endpoint (Law et al. 2009; Weppler and Magnusson 2010).

### Neuromuscular activation and force-generating capacity variables after PST

Twelve weeks of PST did not alter MVC, sEMG RMS, RTD_200_, and EMD. Although the administration of one bout of passive stretching led to acute changes in these parameters (Longo et al. 2014, 2017), the same was not observed after long-term stretching training. Interestingly, despite the PST-induced reduction in passive stiffness, the active neuromuscular and mechanical behaviour of the contracting muscle during MVC was not influenced by passive mechanical changes. Indeed, as hypothesised, a reduction in MTC and GM muscle stiffness would have implicated a delayed force transmission from the muscle to the tendon insertion point, a slower RTD, and, possibly, a reduction in MVC (Longo et al. 2017). However, the present results are not consistent with this potential occurrence. A possible explanation could be provided by two concurring mechanisms: unaltered motor drive towards the muscles involved in PST, and possible unchanged PST-induced tendon stiffness during an active contraction, which are consistent with similar sEMG RMS, RTD_200_ and EMD during MVC after intervention compared to baseline. Similarly, previous studies found no PST-induced changes in maximum muscle strength (Akagi and Takahashi 2014; Konrad and Tilp 2014; Blazevich et al. 2014; Sato et al. 2020) and RTD (Guissard and Duchateau 2004; Blazevich et al. 2014), supporting our outcomes of no effects of passive mechanical alterations on active muscle mechanical characteristics. However, one investigation found a lengthening in EMD during MVC after 8 weeks of knee flexors PST (Minshull et al. 2014). Therefore, further studies are needed to clarify whether or not potential functional PST-induced adaptations are muscle-group-dependent.

### Passive MTC and muscle mechanical properties after PST

Besides stretch tolerance, the PST-induced increase in ROM was accompanied by a decrease in MTC stiffness at Wk12 (~ 17%), an increase in GM muscle elongation (~ 26% at Wk6 and ~ 42% at Wk12), and a decrease in GM muscle stiffness (~ 11% at Wk6 and ~ 16% at Wk12). These results suggest that the increase in ROM could be associated with changes in passive mechanical properties of the structures and tissues surrounding the ankle joint, as well as GM muscle mechanical characteristics. Furthermore, a correlation was found only between the percentage changes in ROM and MTC stiffness after PST, suggesting that the increase in ROM could be explained by a reduction in whole-joint stiffness. Nonetheless, despite a similar time course in ROM and GM muscle stiffness alterations, the lack of correlation between percentage modifications in these two variables highlights that changes in the muscle component of stiffness cannot be the main factor explaining ROM changes over time.

The present results are in accordance with previous studies, in which a decrease in MTC (Kubo et al. 2002; Guissard and Duchateau 2004; Nakamura et al. 2012) and GM muscle stiffness (Blazevich et al. 2014; Nakamura et al. 2017, 2020) have been observed after a PST program, and a correlation between percentage changes in ROM and MTC stiffness has been found (Guissard and Duchateau 2004). In contrast, other studies did not find changes in *triceps surae* MTC (Konrad and Tilp 2014; Blazevich et al. 2014) and GM muscle stiffness (Konrad and Tilp 2014). The discrepancy can be attributable to different training modality, such as intensity, frequency, number of exercises, and overall duration (Freitas et al. 2018). Concerning GM muscle stiffness, another possible explanation could be that PST affected differently the stiffness of the individual *triceps surae* muscles and/or the regional stiffness within each muscle, as observed by shear-wave ultrasound imaging technique (Andrade et al. 2020). Nonetheless, it seems that combining several weeks of training with high weekly exercise frequency, as it has been done in the present study, can lead to MTC and GM muscle stiffness adaptations. Interestingly, between Wk6 and Wk12, we observed a non-significant decrease in muscle stiffness. Hence, it can be speculated that after Wk6, the changes in ROM could be more related to changes in other structures than muscle stiffness.

MTC stiffness could be related to the intrinsic stiffness of muscles, tendons, and connective tissues surrounding the whole MTC and joint (e.g., fascia, ligaments, joint capsule, bursa) and/or to neural mechanisms (Guissard and Duchateau 2004; Nakamura et al. 2012). Previous studies suggested that within the muscle component, a PST programme can change the compliance of the intra- and extracellular structures involved in the passive tension generation during a stretch manoeuvre, particularly: (i) resting filamentary tension, caused by the stretching of the stable cross-link between the actin and myosin filaments (Proske and Morgan 1999); (ii) stretching of the no-contractile proteins, such as titin and desmin proteins (Magid and Law 1985; Trombitás et al. 1998); and (iii) deformation of the connective tissues located within and surrounding the muscle (in particular, the perimysium) (Borg and Caulfield 1980; Rowe 1981). The present results in GM muscle stiffness suggest that mechanical modifications occurred at muscle level possibly involving one or more of the above-mentioned contributors. In contrast, the influence of long-term PST on the tendon component is still controversial. Indeed, as summarised in a recent review (Freitas et al. 2018), previous studies using different PST approaches did not find changes in tendon passive mechanical properties. Finally, despite not being measured in the present investigation, PST-induced neural adaptations explaining changes in MTC stiffness cannot be excluded (Guissard and Duchateau 2004).

### Triceps Surae architecture after PST

Contrary to our hypothesis, the present results demonstrated a lack of architectural adaptations to 12 weeks of PST at all regional levels. The rationale of changes in muscle size and architecture (i.e., longitudinal muscle growth and/or fascicle elongation) was based on several animal studies indicating that joint immobilization in a lengthened position (i.e., stretch) for long periods led to muscle growth and increase in muscle length due to sarcomere addition (Goldspink 1977; Goldspink et al. 1995). Whether or not these adaptations can be obtained also in humans is still controversial. Indeed, Simpson et al. (2017) reported an increase in muscle thickness and GM and GL fascicle length at both mid-belly and MTJ locations after 6 weeks of overloaded static stretching training performed 5 times a week. Similarly, Andrade et al. (2020) observed an increase in mid-belly GM fascicle length after 12 weeks of PST performed 5 times a week, involving similar exercises and training protocol than as used in the present investigation. In contrast, albeit shorter duration (i.e., 3–6 weeks), other studies did not report such adaptations (Nakamura et al. 2012; Konrad and Tilp 2014; Blazevich et al. 2014; Sato et al. 2020). Compared to the study by Simpson et al. (2017), the use of unloaded stretch exercises in the present investigation can explain the differences between studies in the architectural adaptations. However, we do not have a clear explanation for the discrepancy between the present study and the one by Andrade et al. (2020). Hypothetically, this could be ascribed to the use of different ultrasound approaches for determining muscle architecture (i.e., extended field of view versus B-mode ultrasound, respectively). Nonetheless, the present lack of architectural changes extends the results of two recent reviews highlighting the absence of structural and architectural adaptations induced by unloaded static stretching training up to 8 weeks (Freitas et al. 2018; Nunes et al. 2020).

As final consideration, in light of previous studies (Simpson et al. 2017; Andrade et al. 2020), it can be speculated that combining overloaded stretching training with high training volume could lead to possible structural adaptations in the stretched muscles at rest. Future studies are needed to explore this hypothesis.

### Study limitations

This study comes with some limitations. First, the studied population was fairly homogenous (healthy, physically active adults); therefore, generalisation to other populations is limited. Second, possible differences between sexes in the response to PST could not be investigated due to the small sample size; future studies are needed to explore this possible occurrence. Third, it has been recently pointed out that ROM changes can be also explained by nerve stiffness changes due to stretching intervention (Andrade et al. 2020). However, due to the lack of shear-wave elastography-equipped ultrasound, this variable was not measured. Last, the assessment of muscle activity during stretching sessions would have provided more information about the level of muscle activation during elongation. Nonetheless, all of our participants were instructed to reach their own perceived level of maximum stretch within the pain limit, which led to an increase in ROM over the training period.

## Conclusion

The present findings demonstrated that with 12 weeks of PST, the expected increase in ROM and stretch tolerance was accompanied by passive stiffness reduction at both MTC and GM muscle level. Moreover, changes in muscle stiffness occurred already at an earlier stage (Wk6). However, changes in ROM were explained by changes in stretch tolerance and whole MTC stiffness, but not by changes in GM muscle stiffness, indicating that other tissues contributing to the entire joint stiffness could play a major role in determining joint ROM variations. Interestingly, no changes in muscle force-generating capacity variables and architectural features at rest were found. These results provide evidence that the stretching protocol used in the present study induced modifications in the passive mechanical properties of the ankle joint without compromising the plantarflexor muscles force-generating capacity. As an important implication, practitioners can therefore utilize stretching routines chronically without impairing sport or exercise maximum performance. Future studies are needed to assess whether or not PST of longer duration and/or with different protocols could induce resting architectural adaptations.

## Declarations

The Authors would like to thank all the volunteers that participated in the study.

## Abbreviations

95% CI: 95% Of confidence interval
ANOVA: Analysis of variance
ANCOVA: Analysis of co-variance
CTRL: Control group
EMD: Electromechanical delay
*g*: Hedge’s effect size
GL: *Gastrocnemius lateralis* Muscle
GM: *Gastrocnemius medialis* Muscle
ICC: Intraclass correlation coefficient
*L*_f_: Fascicle length
MID: 50% Of muscle belly length
MT: Muscle thickness
MTC: Muscle–tendon complex
MTJ: Myotendinous junction
MVC: Maximum voluntary contraction
PRT_max_: Maximum passive resistive torque
PST: Passive stretchign training
RMS: Root mean square
ROM: Range of motion
RTD_200_: Rate of torque development in the first 200 ms
SEM%: Standard error of measurement as percentage
sEMG: Surface electromyography
SOL: *Soleus* Muscle
STR: Stretching group
Wk12: 12Th week of study protocol
Wk6: 6Th week of study protocol
ΔMTJ_max_: Maximum myotendinous junction displacement
*η*_p_^2^: Partial eta squared
*θ*: Fascicle angle
*ρ*: Spearman’s Rho coefficient
